# Student-AI interaction in computer-assisted consecutive interpreting: patterns and performance

**DOI:** 10.3389/fpsyg.2026.1890808

**Published:** 2026-09-11

**Authors:** Huolingxiao Kuang, Jingyi Li, Yu Weng

**Affiliations:** 1 School of Foreign Languages, Renmin University of China, Beijing, China; 2 Department of Language Science and Technology, The Hong Kong Polytechnic University, Hong Kong, Hong Kong SAR, China

**Keywords:** behavioral patterns, computer-assisted consecutive interpreting, mixed-methods study, student-AI interaction, training effects

## Abstract

While student-AI interaction patterns have been explored in self-paced cross-linguistic tasks such as L2 writing and translation post-editing, little is known about how students interact with AI in bilingual tasks under intense cognitive and temporal constraints. This study uses computer-assisted consecutive interpreting (CACI) as a window to examine student-AI interaction patterns in such high-stakes environments. Twenty-two Chinese-native interpreting trainees, grouped by prior AI training experience, performed bidirectional CACI tasks using AI-enabled systems integrating automatic speech recognition (ASR) and machine translation (MT) features. With data collected from eye-tracking, pen-recording, and voice-recording, the study reveals that: (a) four interpretable interaction profiles emerge: Intensive Engagers (heavy AI reliance), Fast Scanners (scanning-based processing), Traditionalists (minimal AI reliance), and Frequent Switchers (shifts between AI support and handwritten notes); (b) these patterns shift dynamically between the comprehension and production stages of interpreting, while targeted CACI training was associated with more stable, AI-oriented patterns; and (c) students’ interaction patterns during the comprehension stage, but not the production stage, are significantly associated with performance quality. These findings highlight the heterogeneity and variability of student-AI interaction patterns across different stages of complex bilingual tasks and underscore training effects on shaping students’ behaviors and performance.

## 1 Introduction

The integration of artificial intelligence (AI) into language education has presented a double-edged sword. On the one hand, AI systems built upon core functionalities like automatic speech recognition (ASR) and machine translation (MT) have proven powerful learning resources, providing timely and personalized support and enhancing learning efficiency (e.g., Tsai, 2022; Van Lieshout and Cardoso, 2022; Wang H. et al., 2023; Liu and Wang, 2024; Zhong and Ma, 2025). On the other hand, uncritical adoption of these tools raises significant concerns regarding over-reliance (Jose et al., 2025; Ninghardjanti et al., 2025; Zhong and Ma, 2025) and unsustainable learning (Kim and Cho, 2025; Jose et al., 2025; Ninghardjanti et al., 2025). Therefore, to ensure that AI serves as an empowering partner rather than a “free ride,” examining student-AI interaction patterns during actual task performance becomes imperative. However, recent studies examining the behavioral patterns of student-AI interaction in different language mediation settings remain mostly confined to self-paced learning tasks, where learners are allowed to pause, consult, and revise AI-generated output in an iterative manner (e.g., Wang X. et al., 2023; Ji et al., 2024; Cai and Tian, 2026; Li et al., 2026). Consequently, student-AI interaction patterns remain relatively underexplored in tasks characterized by high temporal pressure and intense cross-linguistic switching demand.

Interpreting is widely recognized as one of the most complex bilingual skills, characterized by rapid interlingual transfer under tight time constraints (Yu and Dong, 2022) and inherent multitasking that requires coordinated cognitive efforts such as listening and analysis, production, memory, and coordination (Gile, 2002). These cognitive demands distinguish interpreting from other second-language-mediated tasks, and its real-time, high-stakes nature offers a distinctive window to examine student-AI interaction dynamics.

These demands have motivated the development of computer-assisted interpreting (CAI), a family of tools, including AI-driven technologies such as ASR and MT, that support interpreters at work. In contemporary CAI, interpreters often work with real-time ASR transcripts for source speech comprehension and MT references for target speech production. While offering aids, it requires dynamic coordination between internal cognitive processing and external AI consultation (Fantinuoli, 2023), imposing an additional Human-Machine Interaction Effort (Gile, 2025). Unlike translation tasks, the fleeting nature of spoken language allows for no “pause-and-ponder”; decisions to trust or reject AI input must be made under intense time pressure. Recent studies suggest that interpreting trainees approach AI support in highly varied ways, largely due to their developing expertise and the lack of systematic, established strategies for engaging with AI, which leads to notable effects on interpreting performance (e.g., Wang and Wang, 2019; Defrancq and Fantinuoli, 2021; Sun et al., 2021; Tan et al., 2025; Wang et al., 2025). Coupled with instructors’ insufficient knowledge about student-AI interaction patterns, this prevents evidence-based curriculum design for AI-assisted interpreting (Guo et al., 2024).

Against this backdrop, we examine student-AI interaction patterns in complex bilingual tasks—using computer-assisted consecutive interpreting (CACI) as the study context—across four interconnected dimensions: (1) characterizing fine-grained interaction behaviors under varying AI-support conditions, (2) investigating pattern shifts across the input (listening/note-taking) and output (reformulating/note-reading) stages of interpreting (Gile, 2009), (3) examining how formal training modulates interaction patterns, and (4) exploring associations between pattern adoption and interpreting performance. Altogether, we aim to contribute empirical insights into effective AI integration for interpreting and broader bilingual processing tasks, contributing to practical design of CAI tools, pedagogical support for interpreter training, and future research on human-AI collaboration.

## 2 Research background

Existing empirical research on CAI has primarily focused on the impact of technology integration on the product and process of interpreting. Product-oriented studies have generally reported gains in interpreting accuracy, but not necessarily in fluency (e.g., Desmet et al., 2018; Wang and Wang, 2019; Defrancq and Fantinuoli, 2021; Cheung and Li, 2022; Ünlü and Doğan, 2024; Yu and Shao, 2025). More recent studies have shifted attention to the interpreting process, examining AI tools’ potential to reduce interpreters’ cognitive load (e.g., Li and Chmiel, 2024; Yu and Shao, 2025) and psychological stress (e.g., Olalla-Soler et al., 2023), while also examining interpreters’ perceptions of these tools (e.g., Su and Li, 2024; Wang et al., 2025; Zhang et al., 2026). Despite these insights, three important research gaps remain: (1) a predominant focus on simultaneous interpreting (SI), leaving consecutive interpreting (CI) relatively underexplored; (2) a participant profile dominated by student interpreters with limited or no CAI training background; and (3) a research focus largely on product improvement or process ease (e.g., cognitive load), rather than on the student–AI interaction itself.

The few prior CI studies converge on a consistent trade-off. Wang and Wang (2019) tested MT support from Google Translate, Ünlü and Doğan (2024) incorporated ASR, MT, and other support via Sight-Terp, and Yu and Shao (2025) examined Otter.ai-generated ASR transcripts. All three studies reported improved interpreting accuracy or fidelity in tool-assisted conditions. However, these gains were frequently accompanied by reduced fluency, evidenced by more disfluency markers, lower delivery rates, and longer task completion times. Yu and Shao (2025) proposed that this performance trade-off arises because AI-induced comprehension and memory benefits are offset by additional coordination and processing demands when managing external input, an assumption supported by their finding that self-reported task load remained similar in AI-supported versus conventional settings. Complementing this pattern of accuracy-fluency trade-offs, findings from Zhang et al. (2026) point to a further quality dimension shaped by directionality: with ASR and MT support (provided through iFlytek machine interpreting system), interpreting into the A language (L1) yielded superior performance and lower cognitive load compared to into-B (L2). This suggests that technological affordances offer limited mitigation of the fundamental processing asymmetries between into-A and into-B interpreting directions. Overall, existing empirical research on CACI reports mixed effects of AI support on interpreting quality while also indicating possible cognitive reconfiguration as interpreters adapt to technology integrated into the workflow.

CAI research broadly agrees that targeted training shapes how interpreters use AI support (Defrancq and Fantinuoli, 2021; Guo et al., 2023; Li and Chmiel, 2024), yet what counts as training varies notably regarding its duration and pedagogical approach: from 2 hours (Frittella and Rodríguez, 2022) to over 2 months (Chen and Kruger, 2023, 2024a, 2024b), delivered through classroom demonstrations (Tan et al., 2025), theory-and-practice seminars (Restuccia, 2024), and self-paced online modules (Frittella and Rodríguez, 2022). Only three of these studies on CI provided structured training. Chen and Kruger (2023, 2024a, 2024b) proposed a novel CACI workflow in which interpreters respeak the source speech to trigger ASR transcription, then draw on the resulting transcript and its MT rendering when producing the target speech. They developed and evaluated a 10-week blended training program for this mode. Post-training results showed that students achieved overall higher interpreting quality in CACI than in conventional CI for both directions, though fluency gains and reduced cognitive load appeared only from L1 into L2. Structured training and directionality therefore both condition CACI effects.

Yu and Shao (2025) provided participants with five two-hour sessions (spanning 10 weeks in total) covering ASR application and interpreting practice. Beyond quantitative findings, interviews revealed that participants struggled to integrate ASR transcripts with note-taking—a core conventional CI component. The challenge lies in coordinating visual attention between the screen-displayed ASR transcript and paper-based notes, which introduces frequent attentional switches that counter any cognitive load reductions ASR might provide, thereby undermining its potential efficiency benefits. Unlike the technology-as-assistance approaches, Chang and Yamada (2026) examined GenAI as a learning tool, integrated into preparation, inter-attempt support, and post-performance feedback stages of interpreting. The eight-week study revealed students’ progression from passive tool use to metacognitive engagement, yet this focused training context differs fundamentally from real-time interpreting demands, limiting the transferability of learning gains to authentic interpreting scenarios.

Direct evidence on the interpreter-AI interaction itself is scarce. Within interpreting, the closest comes from Jiang (2025), who observed caption-viewing behavior in ASR-supported SI and identified three patterns: (1) frequent caption viewing, reflecting difficulty to balance speaking, reading, and listening simultaneously; (2) infrequent but prolonged viewing, indicating heavy reliance on captions; and (3) moderate frequency and duration, representing strategic integration between independent processing and ASR consultation. In written translation, Cai and Tian (2026) quantified student-AI interaction through process metrics such as AI engagement time and frequency, identifying distinct interaction profiles that varied significantly in translation quality. This finding underscores that interaction patterns, and not the availability of AI support alone, profoundly shape translation outcomes.

However, Cai and Tian’s (2026) findings do not transfer directly to interpreting. In self-paced translation, human-AI interaction is often mediated through explicit conversational turns, the extreme temporal constraints of interpreting necessitate a distinct, implicit interaction where interpreters make trust-or-not decisions in real time, potentially inducing additional cognitive demands and workflow complexity (e.g., Du and Wang, 2025; Li and Chmiel, 2024; Peng et al., 2025; Zhang and Xie, 2025). Interpreting studies that invoke interaction patterns to explain their findings typically infer them from post-task interviews or unsystematic observations (e.g., Defrancq and Fantinuoli, 2021; Frittella and Rodríguez, 2022; Wang et al., 2025; Zhang and Xie, 2025). Jiang (2025) aside, no interpreting research has derived interaction profiles from quantitative process data.

To address these gaps, the present study examines student-AI interaction patterns in CACI, a prototypical high-pressure bilingual task that introduces unique Human-Machine Interaction Effort based on Gile’s (2025) updated Effort Model. The embedded time pressure and bilingual coordination demand of CACI, coupled with its stage-based structure (input and output), make interaction dynamics observable under shifting task demands. During the input stage, where interpreters conventionally rely on note-taking for source information storage and comprehension, real-time ASR transcripts and MT references provide externalized support that may reconfigure the trade-off between note-taking and memory. During the output stage, students must deliver accurate and fluent renderings through a coordinative use of handwritten notes and ASR/MT outputs. This workflow enables tracing how students distribute attention and integrate AI support across interpreting stages. To capture interaction patterns across diverse realistic conditions, we include two participant groups (with and without CACI training experience), two interpreting directions (from Chinese to English and vice versa), and three AI configurations (low-quality ASR, high-quality ASR, and ASR-plus-MT) in the investigation. Altogether, this study aims to provide a process-oriented account of student-AI interaction patterns in CACI, investigating their shifts across different interpreting stages and following targeted training. By typologizing student-AI interaction in this time-pressured bilingual task, this study seeks to inform interpreter education and broader complex language skill acquisition where effective AI integration hinges on rapid, selective, and accountable AI use.

## 3 Method

### 3.1 Participants

Twenty-seven native Chinese-speaking Master’s students were initially recruited from four interpreting programs in Beijing. After excluding the participants with incomplete eye-tracking data, 22 participants were included in the analysis. Participants were assigned to two groups based on whether they had received formal AI-assisted interpreting training. Group A (*n* = 11; 11 female) had completed systematic CACI training and was recruited from a single institution to standardize that exposure. The training was delivered through a 16-week compulsory course in an interpreting program. At the time of data collection, the course had been offered for 2 years and therefore followed a relatively well-established instruction design. It focused on introducing core concepts of AI-assisted interpreting and providing opportunities for students to practice with various AI tools, adhering to its pre-defined objectives and syllabus. Crucially, instruction emphasized exploratory engagement and critical reflections rather than prescribing specific interpreting workflows.

Group B (*n* = 11; 8 female, 3 male) had no systematic AI-assisted interpreting training and was recruited from three other institutions. Potential confounds related to institutional differences (e.g., curricula variation, teaching practices, student characteristics) were addressed or mitigated in several ways. All participants, across both groups, were enrolled in the interpreting stream of two-year Master of Translation and Interpreting (MTI) programs at top-tier universities. They had all completed first-year core coursework covering both consecutive and simultaneous interpreting, providing them with comparable foundational training and curricular preparation. Furthermore, as detailed in Table 1, the two groups had comparable self-reported English proficiency and general interpreting training experience. Ethical approval was obtained prior to the study.

**Table 1:** Participant details.

Category	Group A with CACI training (*n* = 11)	Group B no CACI training (*n* = 11)
Age	24.45 (SD = 2.02)	23.27 (SD = 0.90)
English proficiency: TEM8 scores^1^	77.45 (SD = 4.30)	78.29 (SD = 4.42)^2^
Interpreting training time (years)	2.14 (SD = 1.30)	2.18 (SD = 0.57)
Note-taking training time (years)	1.82 (SD = 1.23)	1.32 (SD = 0.75)

^1^The Test for English Majors Grade Eight (TEM8) is a national English test in China. ^2^The TEM8 score for Group B is based on 7 students. The remaining 4 students without TEM8 scores had English proficiency at CEFR C1 level or above.

### 3.2 Materials

The study reported here belongs to a larger project on computer-assisted language learning, with eight speeches developed in total as experimental stimuli. For this study, three Chinese and three English speeches were used for low-quality ASR, high-quality ASR, and ASR-plus-MT task conditions.

Initial speech drafts were generated using ChatGPT-4o based on two typical official conference addresses (one Chinese, one English). A detailed prompt directed ChatGPT to create speeches of 110 words in English and 165 words in Chinese (given the 1.5:1 Chinese character-to-English word ratio in Sun et al., 1985). This length (approximately one-minute in duration) aligned with students’ common classroom practices and interpreting proficiency test segments. The speeches closely mirrored the reference texts’ content structure, tone, and linguistic style. Subsequently, two interpreting instructors selected and refined eight speeches for logical coherence and appropriate expressions, ensuring both English and Chinese versions were comparable in complexity while varying only in content and source language. A five-student pilot study confirmed that all speeches conveyed a substantial amount of information that necessitated note-taking for successful interpretation. While Chinese and English materials were controlled for length, pilot participants found that the Chinese texts presented a higher overall information load. The instructors therefore recalibrated Chinese text length to approximately 135 words. The finalized texts were reviewed for information load and readability, with minor adjustments to ensure balance and consistency across all materials (Table 2).

**Table 2:** Details of speeches used in this study.

Speech	AI mode	Word count	Zipf frequency	Idea density	Sentence count	Sentence length	Dependency distance	Duration	ASR accuracy rate	MT BLEURT-20 score
English 1	Low-quality ASR^1^	107	4.091	0.505	8	13.375	2.398	1′21”	0.851	n/a
English 2	High-quality ASR	104	4.006	0.500	9	11.556	2.273	1′16”	0.990	n/a
English 3	ASR plus MT	125	3.821	0.508	9	13.889	2.314	1′28”	0.984	0.602
Chinese 1	Low-quality ASR^2^	135	4.552	0.504	11	12.273	2.537	1′25”	0.852	n/a
Chinese 2	High-quality ASR	135	4.369	0.511	10	13.500	2.659	1′24”	0.993	n/a
Chinese 3	ASR plus MT	136	4.620	0.507	12	11.333	2.321	1′18”	0.978	0.656

^1,2^We targeted 85% ASR accuracy for the low-quality condition, as rates below 90% impair interpreting performance (Li and Chmiel, 2024), by introducing evenly-distributed recognition errors across word class (nouns, verbs, adjectives) and context dependency.

To complement the pilot feedback, we further assessed the materials using objective measures of lexical frequency, information density, and syntactic complexity. These measures were intended to establish operational comparability for the experimental tasks. English idea density was calculated using CPIDR 5.0, while Chinese propositions were manually coded through HanLP-assisted semantic-role analysis using rules informed by Chinese Proposition Bank 3.0 (CPB3; LDC2013T13). Lexical difficulty was indexed by the mean Zipf frequency of nouns, verbs, adjectives, and adverbs, using SUBTLEX-US (Brysbaert and New, 2009) for English and SUBTLEX-CH (Cai and Brysbaert, 2010) for Chinese. Syntactic complexity was indexed by dependency distance under the Universal Dependencies framework. Detailed calculation and preprocessing procedures are provided in Appendix A, and the results are reported in Table 2.

To ensure consistent audio quality and speaker style, all materials were converted into audio via www.text-to-speech.com using a standard accent voice and uniform speech rate. No participants questioned the authenticity of the synthesized voices in neither pilot nor the formal study. To avoid variability of ASR outputs across participants caused by network issues or model fluctuations, the AI outputs for each speech was pre-recorded. During the experiment, the researcher played the videos where the ASR and MT results unfolded dynamically as the audio played. The ASR accuracy rate (calculated as the percentage of correctly-transcribed words) and MT quality (indicated by BLEURT-20 score) were carefully matched between the corresponding English and Chinese speeches for each AI mode (Table 2).

At the discourse level, the six speeches shared a comparable four-point enumerative conference-speech macrostructure. Each speech began with an audience address and a brief topic introduction, explicitly previewed four main points, and then developed four numbered thematic units. Each unit introduced a focal action or topic and provided explanatory, consequential, or benefit-related information. None of the speeches contained an extended narrative, counterargument, or separate concluding section, with closure generally incorporated into the final unit. The materials were therefore considered comparable in overall macrostructure.

### 3.3 Experiment set-up and procedures

**Figure 1:**
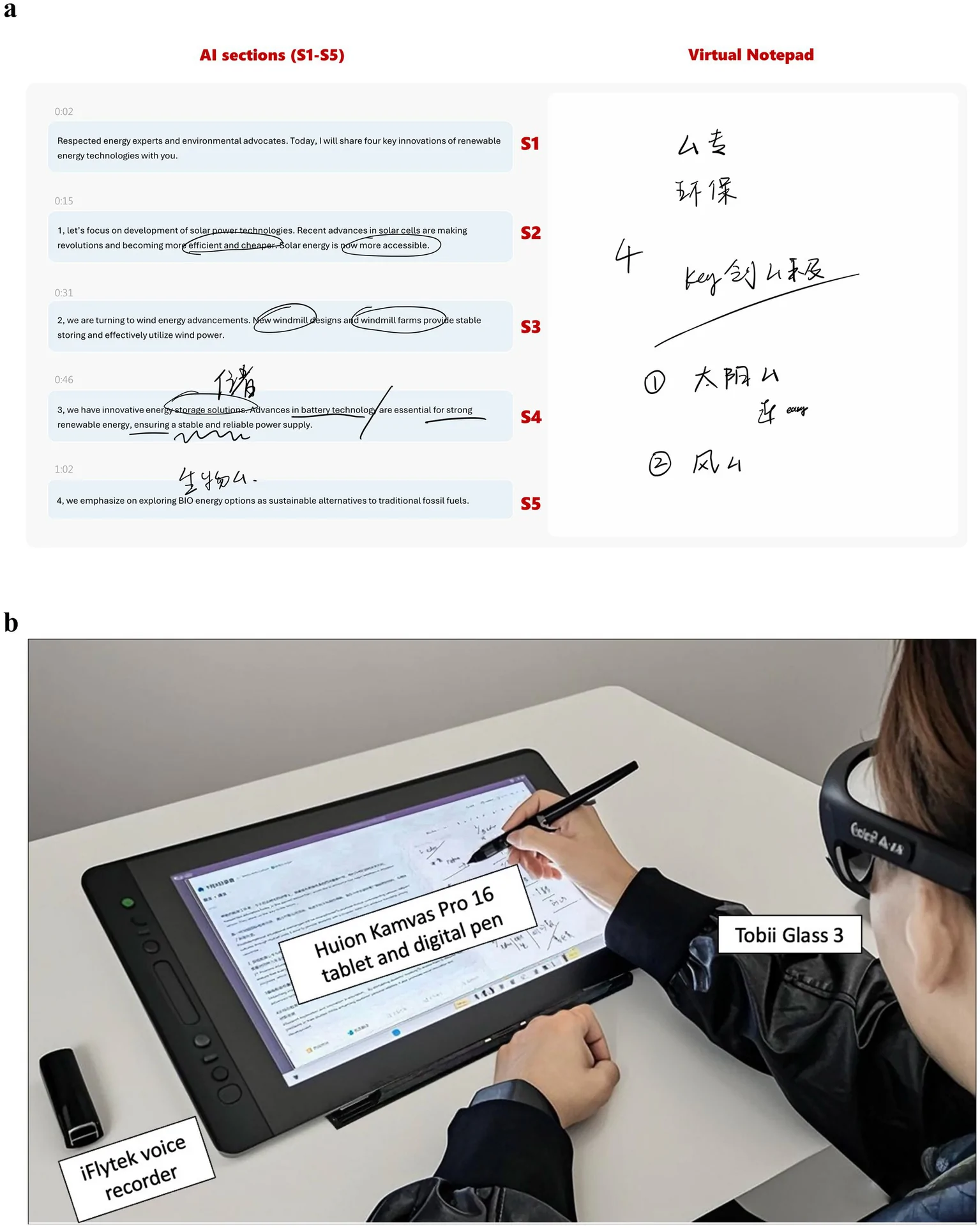
Interface of iFlytek **(a)** and experimental setup **(b)** of the study.

We used the iFlytek platform, which features an interface including an ASR/MT display area on the left and a virtual notepad on the right, to facilitate the experiment (Figure 1a). The platform automatically divided speeches into segments based on transitional keywords (e.g., “first of all”, “in conclusion”). As the materials were structured into five sections with such transitional expressions, the platform produced identical segment structures across all tasks, ensuring structural and visual uniformity across different tasks.

Participants took notes using a digital pen on a Huion Kamvas Pro16 digital drawing tablet. The tablet offers a 1,920 × 1,080 pixel screen resolution and accompanies a foldable stand allowing for a comfortable 45° viewing angle. During the interpreting tasks, the participants were free to write anywhere on the screen, including the ASR/MT display area.

Participants’ eye and pen movements were recorded during both the input (listening and note-taking) and output (note-reading and speech production) stages of interpreting via a Tobii Glass 3 eye-tracker (sampling rate: 100 Hz) and a Huion digital pen. The interpretation outputs were recorded using an iFlytek recording pen to ensure accurate synchronization with system logs (Figure 1b).

The experiment began with participants signing an informed consent and adjusting the eye-tracker lenses to match their vision prescriptions. In the warm-up session, participants completed one interpreting task in the ASR-assisted mode and another in the ASR-plus-MT assisted mode, followed by a time-free break. The main experiment comprised three tasks per direction (i.e., E-C and E-C) presented in counterbalanced order. Following each task, participants completed a brief questionnaire assessing their perceptions of the AI tools. After interpreting, cued retrospection interviews were conducted to discuss their experience with different interpreting modes, explain their note-taking choices, and reflect on problem-solving strategies. The experiment lasted 70–80 min.

### 3.4 Measures

Eye movements consists primarily of fixations and saccades. Fixations are relatively static states of the eyes, typically lasting around 200–300 milliseconds, where visual information is acquired and processed; saccades are rapid eye movements between fixations, typically lasting between 20 and 200 milliseconds (Carter and Luke, 2020). While fixations are commonly believed to indicate where cognitive processing is occurring and the intensity of that processing, saccades are crucial for repositioning the eyes to bring new visual information into focus. This provides the fundamental basis for deriving relevant eye-tracking indicators to examine participants’ AI interaction patterns in this study. Meanwhile, established process-oriented translation and interpreting research employing eye-tracking and screen recording have demonstrated that metrics such as interaction time, fixation count per second, and regression measures are able to capture key aspects of translators’ and interpreters’ behavior (e.g., Doherty et al., 2022; Du and Wang, 2025; Cai and Tian, 2026; Yuan and Wang, 2023).

Accordingly, we adopted six eye-tracking measures to quantify participants’ interactions with AI tools for the subsequent cluster analysis (see Table 3). With areas of interest (AOIs) drawn on the AI display area (for ASR and MT references) and the notepad area (for handwritten notes), AI reading proportion and count of switches between the two AOIs were used to capture the distribution and dynamics of overt visual attention. Regression saccade rate and saccade amplitude characterize scanning behavior, with the former indicating the frequency of backward eye movements for information reanalysis and the latter reflecting the spatial extent of visual exploration. Fixation frequency and deep fixation proportion index processing density and depth, revealing whether interpreters engaged in rapid, shallow scanning or slower, more deliberate cognitive processing.

**Table 3:** Details of the measures.

Data source	Measures	Operationalization
Eye-tracking	AI reading proportion	The proportion of fixation duration allocated to the AI display area / the total fixation duration of the corresponding input or output stage
	Regression saccade rate	Regressive saccade^1^ count / total saccade counts of the corresponding stage
	Saccade amplitude	Average saccade amplitude of the corresponding stage
	Fixation frequency	Fixation count per minute of the corresponding stage
	Deep fixation proportion	Number of fixations > 250 ms / total fixation counts of the corresponding stage
	Switch count	Number of switches between AI and notepad of the corresponding stage
Pen-recording	Note count	Number of notes taken on the AI display area and the notepad area respectively
	Note-taking duration	Total handwriting time during note-taking on the AI display area and the notepad area respectively
Voice-recording (temporal aspect)	Target speech duration	Target speech duration in milliseconds
	Pause count	Number of pauses at 250 ms threshold
	Pause duration proportion	Total pause duration (at 250 ms threshold) / target speech duration
	Filler count	Number of filler words
	Disfluency count	Number of disfluency markers
Voice-recording (quality aspect)	Information completeness	The accuracy and completeness of the conveyed message content
	Fluency of delivery	The smoothness and continuity of speech production
	Target language quality	The grammatical correctness and idiomaticity of the output language

^1^Regressive saccades (backward eye movements) were operationalized as saccades with directional angles of 90–225 degrees, filtered automatically and manually verified.

To investigate note-taking behavior, process-oriented (note-taking duration) and product-oriented (note counts) measures were derived from pen-recordings following previous note-taking research (Chen, 2020; Kuang and Zheng, 2022). These measures were calculated, respectively, for the left AI support area and the right notepad area to identify whether students directly interacted with the AI outputs or engaged in relatively independent note-taking.

Interpreting products were examined from two aspects: temporal features and interpretation quality. The former was assessed through overall length (target speech duration), hesitation frequency (silent pause count), hesitation intensity (silent pause duration proportion), and disruptions within ongoing speech (including filled pauses and disfluencies such as repetitions, false starts, and repairs) based on previous studies (Yu and Van Heuven, 2017). The later was investigated through expert ratings by two experienced interpreters across three dimensions: information completeness (InfoCom), fluency of delivery (FluDel), and target language quality (TLQual) (Han, 2019), using an 8-point scale distributed across four performance bands. Raters first categorized performances into a band before assigning specific scores. Inter-rater reliability coefficients of the assessments (0.774 ~ 0.854) exceeded the acceptable threshold of 0.70.

### 3.5 Statistical analysis

K-means cluster analysis was used to identify the fundamental interaction patterns across the two interpreting stages. For cross-stage comparability, each task at each stage was treated as a separate observation and entered a unified dataset, yielding 264 stage-level observations (132 input-stage and 132 output-stage observations) nested within 22 participants. Each observation was represented by the same the six eye-tracking measures (see section 3.4), which were standardized to *Z*-scores to ensure equal weighting (Jain, 2010). Clusters were defined on this merged data, and the resulting cluster centers were used to assign cluster membership for samples from each stage separately. This methodology ensures that cluster labels represent the same behavioral patterns across both stages, enabling meaningful interpretation of pattern consistency and transitions. The optimal number of clusters (k) was determined using multiple convergent criteria: silhouette coefficient (Rousseeuw, 1987), Calinski-Harabasz index (Caliński and Harabasz, 1974), and Gap statistic (Tibshirani et al., 2001). A combined score was calculated by normalizing each criterion to [0,1] and averaging across the three criteria, with the k value yielding the highest combined score (*k* = 4) selected as optimal. Internal validity metrics, cluster centroids, principal component analysis projections of the four clusters, and the cluster determination details are provided in Appendix B.

To examine whether the identified clusters differed on the clustering measures themselves, one-way ANOVAs or Kruskal–Wallis tests (depending on distribution normality) were conducted. Post-hoc pairwise comparisons were performed using Tukey’s HSD for parametric analyses or Dunn’s test with Bonferroni correction for non-parametric analyses. All statistical analyses were conducted using Python (version 3.9) with *scipy*, *scikit-learn*, and *scikit-posthocs* packages.

## 4 Results

### 4.1 Interaction patterns in CACI

**Figure 2:**
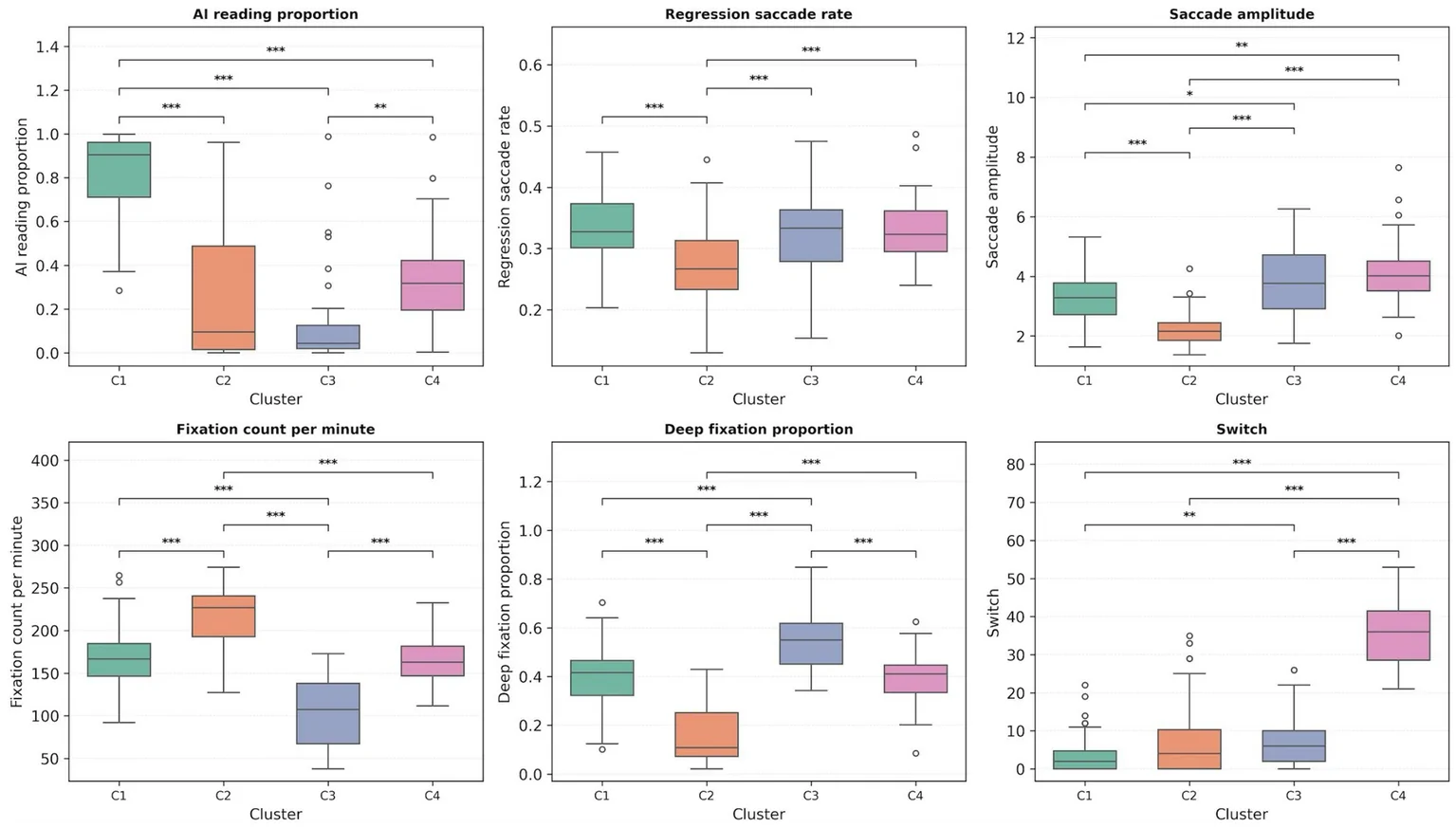
Eye-tracking profiles of student–AI interaction patterns: Intensive Engagers (C1), Fast Scanners (C2), Traditionalists (C3), and Frequent Switchers (C4).

The cluster analysis based on the full dataset (264 stage-level samples) yielded four interpretable interaction patterns, with significant differences observed in all measures across the clusters (Table 4). The boxplots in Figure 2 illustrate the distribution of the eye-tracking measures across the clusters, and Table 5 presents the distribution of note-taking measures.

**Table 4:** Descriptive and inferential statistics for eye-tracking measures by cluster.

Measure	Cluster 1	Cluster 2	Cluster 3	Cluster 4	F/H Statistics
Sample count					
Overall (*n* = 264)	98 (37.12%)	76 (28.79%)	54 (20.45%)	36 (13.64%)	N/A
Input (*n* = 132)	51 (38.64%)	26 (19.70%)	43 (32.58%)	12 (9.09%)	N/A
Output (*n* = 132)	47 (35.61%)	50 (37.88%)	11 (8.33%)	24 (18.18%)	N/A
AI reading proportion					
Overall	0.83 (0.17)^a^	0.27 (0.32)^b,c^	0.12 (0.19)^b^	0.35 (0.22)^c^	H = 152.31***
Input	0.93 (0.07)^a^	0.33 (0.41)^b^	0.11 (0.20)^b^	0.302 (0.08)^b^	H = 83.78***
Output	0.71 (0.16)^a^	0.24 (0.26)^b^	0.16 (0.18)^b^	0.37 (0.26)^b^	H = 66.49***
Regression rate					
Overall	0.33 (0.05)^a^	0.27 (0.07)^b^	0.32 (0.06)^a^	0.33 (0.05)^a^	*F* = 16.04***
Input	0.34 (0.05)^a^	0.24 (0.06)^b^	0.32 (0.07)^a^	0.34 (0.06)^a^	*F* = 18.00***
Output	0.33 (0.06)^a^	0.29 (0.06)^b^	0.34 (0.06)^a,b^	0.33 (0.05)^a,b^	H = 12.56**
Saccade amplitude					
Overall	3.24 (0.79)^a^	2.20 (0.52)^b^	3.87 (1.13)^c^	4.14 (1.09)^c^	H = 119.53***
Input	3.43 (0.81)^a^	1.99 (0.54)^b^	3.98 (1.17)^a,c^	4.78 (1.39)^c^	H = 58.59***
Output	3.03 (0.73)^a^	2.31 (0.48)^b^	3.43 (0.87)^a,c^	3.82 (0.75)^c^	H = 54.25***
Fixation frequency					
Overall	165.43 (34.38)^a^	218.61 (33.75)^b^	103.36 (37.50)^c^	163.40 (29.11)^a^	H = 149.56***
Input	159.84 (41.78)^a^	221.81 (43.59)^b^	98.81 (38.55)^c^	150.93 (36.46)^a^	H = 67.02***
Output	171.50 (22.87)^a^	216.95 (27.67)^b^	121.17 (27.84)^c^	169.63 (23.06)^a,c^	H = 71.33***
Deep fixation proportion					
Overall	0.39 (0.12)^a^	0.17 (0.12)^b^	0.56 (0.12)^c^	0.39 (0.11)^a^	H = 150.77***
Input	0.41 (0.14)^a^	0.15 (0.14)^b^	0.56 (0.13)^c^	0.38 (0.09)^a^	H = 66.99***
Output	0.378 (0.099)^a^	0.174 (0.105)^b^	0.535 (0.082)^c^	0.387 (0.120)^a,c^	H = 73.514***
Switch count					
Overall	3.30 (4.25)^a^	7.00 (8.80)^a,b^	7.65 (6.90)^b^	36.03 (9.45)^c^	H = 105.01***
Input	3.47 (4.23)^a^	7.69 (10.58)^a,b^	7.77 (7.00)^b^	39.50 (10.53)^c^	H = 40.63***
Output	3.11 (4.32)^a^	6.64 (7.81)^a^	7.18 (6.81)^a^	34.29 (8.56)^b^	H = 63.12***

Values are presented as mean (standard deviation). H values denote Kruskal–Wallis test statistics and F values denote one-way ANOVA test statistics. ***p < 0.001, **p < 0.01, *p < 0.05. Superscript letters indicate the results of Dunn’s/Tukey’s HSD *post hoc* comparisons across clusters; means in the same row sharing the same superscript letter do not differ significantly (*p* ≥ 0.05), whereas means with different superscript letters differ significantly at *p* < 0.05. These apply to all subsequent tables.

**Table 5:** Cluster comparison of note-taking measures.

Area	Measure	Cluster 1	Cluster 2	Cluster 3	Cluster 4	Statistics
AI display	Note count	8.61 (9.98)^a^	2.35 (3.36)^b^	0.05 (0.31)^b^	0.08 (0.29)^b^	73.11***
	Note-taking duration	11,055 (18980)^a^	1,071 (2310)^b^	6 (41)^b^	89 (308)^b^	76.86***
Notepad	Note count	2.18 (3.85)^a^	40.54 (27.45)^b^	48.88 (16.26)^b^	40.83 (11.24)^b^	69.23***
	Note-taking duration	2,509 (4185)^a^	39,042 (28027)^b^	69,608 (77530)^b^	49,392 (16346)^b^	70.28***

Note-taking duration is measured in milliseconds.

Cluster 1 is marked by the highest AI reading proportion and the lowest switch count between AI and notepad areas in both stages of interpreting. Accordingly, it features with the highest note count and longest note-taking duration within the AI display area and the lowest values for these measures in the notepad area. These together reflect substantial attention to the AI outputs and limited attention shifts during interpreting. Other eye-tracking measures remain moderate except for relatively high regression saccade rates, which suggest a certain level of reanalysis during AI-output reading. Collectively, these features portray this cluster as Intensive Engagers who primarily interact with AI output through reading and direct annotation, largely bypassing traditional notepad usage.

Cluster 2 features the highest fixation frequency, lowest regression rates, saccade amplitudes and deep fixation proportions, indicating visually dense but shallow, short-range, and linear scanning. Students show highly dispersed AI reading distributions, combined with low-to-moderate switches between the two AOIs, suggesting the co-existence of both AI- and self-reliant approaches within this cluster. Regarding note-taking behavior, despite no significant differences with Cluster 3 or Cluster 4, this cluster shows the second-highest note count and note-taking duration in the AI display area (though still very limited), and the second-lowest values for both measures in the notepad area. This behavioral configuration points to a fragmented interaction style characterized by rapid, localized scanning with frequent short fixations rather than sustained attention on visual elements. Accordingly, this pattern is named as Fast Scanners.

Cluster 3 maintains a workflow closest to traditional consecutive interpreting, with the least reading proportion, note count and note-taking duration in the AI area, but the highest note count and note-taking duration in the notepad area. With the lowest fixation frequency but the highest deep fixation proportion, this cluster features selective but cognitively demanding engagement with limited visual input. Saccade amplitudes are relatively long, and regression saccade rates are moderate, suggesting broad visual movements and certain reanalysis. Switching count between areas is also low, consistent with their behavioral pattern of reliance on memory and self-generated notes. This pattern can be marked as Traditionalists.

Cluster 4 is distinguished by the highest switch frequency. AI reading proportion is moderate, while regression rate and saccade amplitudes are high, suggesting balanced engagement with AI references and self-generated notes, coupled with broad visual exploration across the interface. Levels of fixation frequency and deep fixation proportions indicate moderate processing depth. This pattern reflects an intensive integration strategy, in which interpreters actively coordinate multiple visual resources (AI text and notes) through frequent visual transitions. It is thus tagged as Frequent Switchers.

### 4.2 Cross-stage pattern transitions

To examine behavioral consistency, we compared cluster memberships at the input and output stages for each task. Results showed that 58.3% of task samples maintained the same pattern across stages, while 41.7% exhibited pattern shifts.

**Figure 3:**
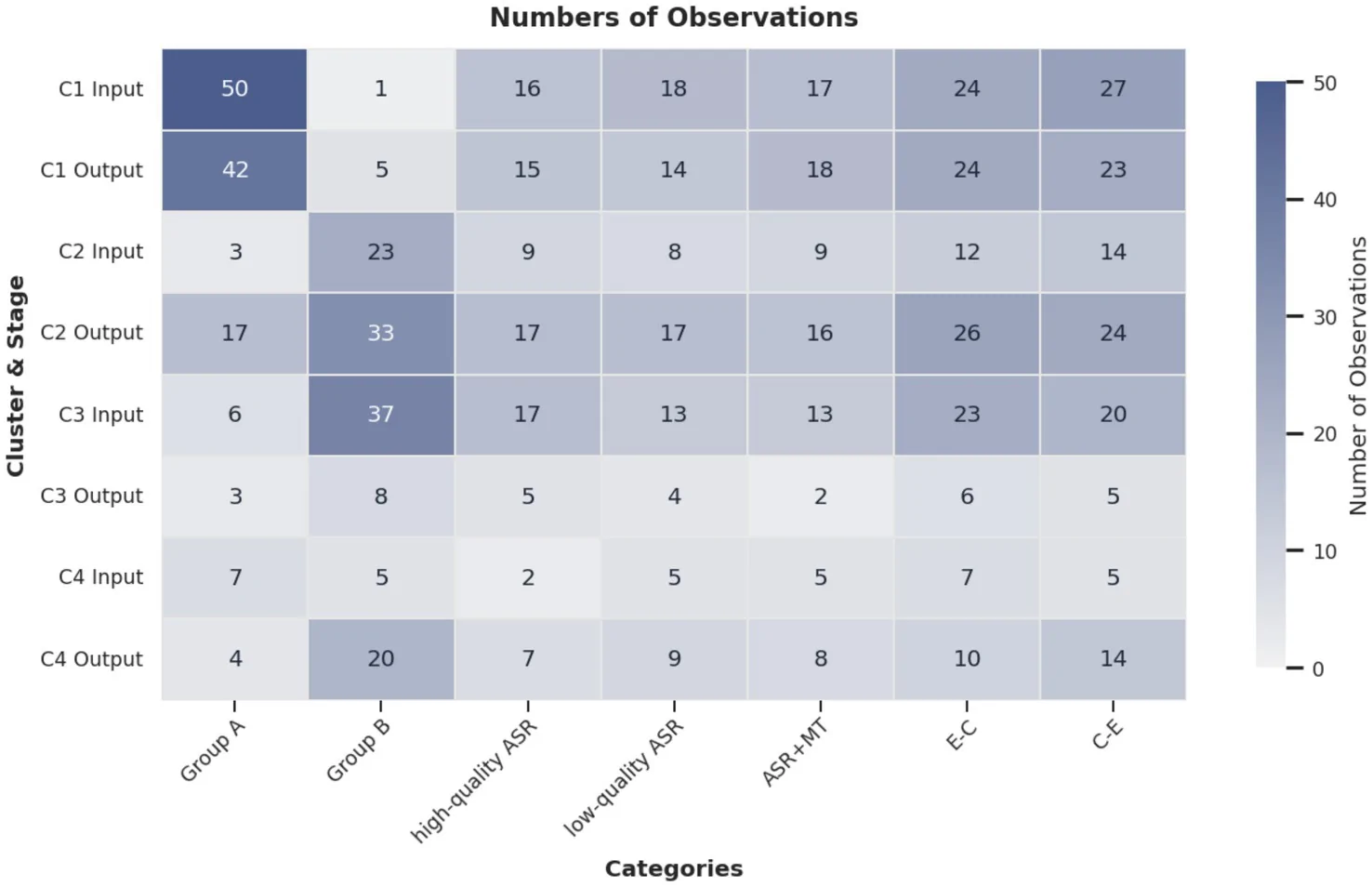
Pattern distributions across participant groups, task modes, and interpreting directions by cluster and stage.

Specifically, Cluster 2 (Fast Scanners) demonstrated the highest consistency rate at 96.2%, followed by Cluster 1 (Intensive Engagers) at 74.5% and Cluster 4 (Frequent Switchers) at 50%. Cluster 3 (Traditionalists) was highly shifty, with only 18.6% of participants maintaining this pattern consistently. The pattern distributions by group, task mode and interpreting direction are presented in Figure 3.

**Figure 4:**
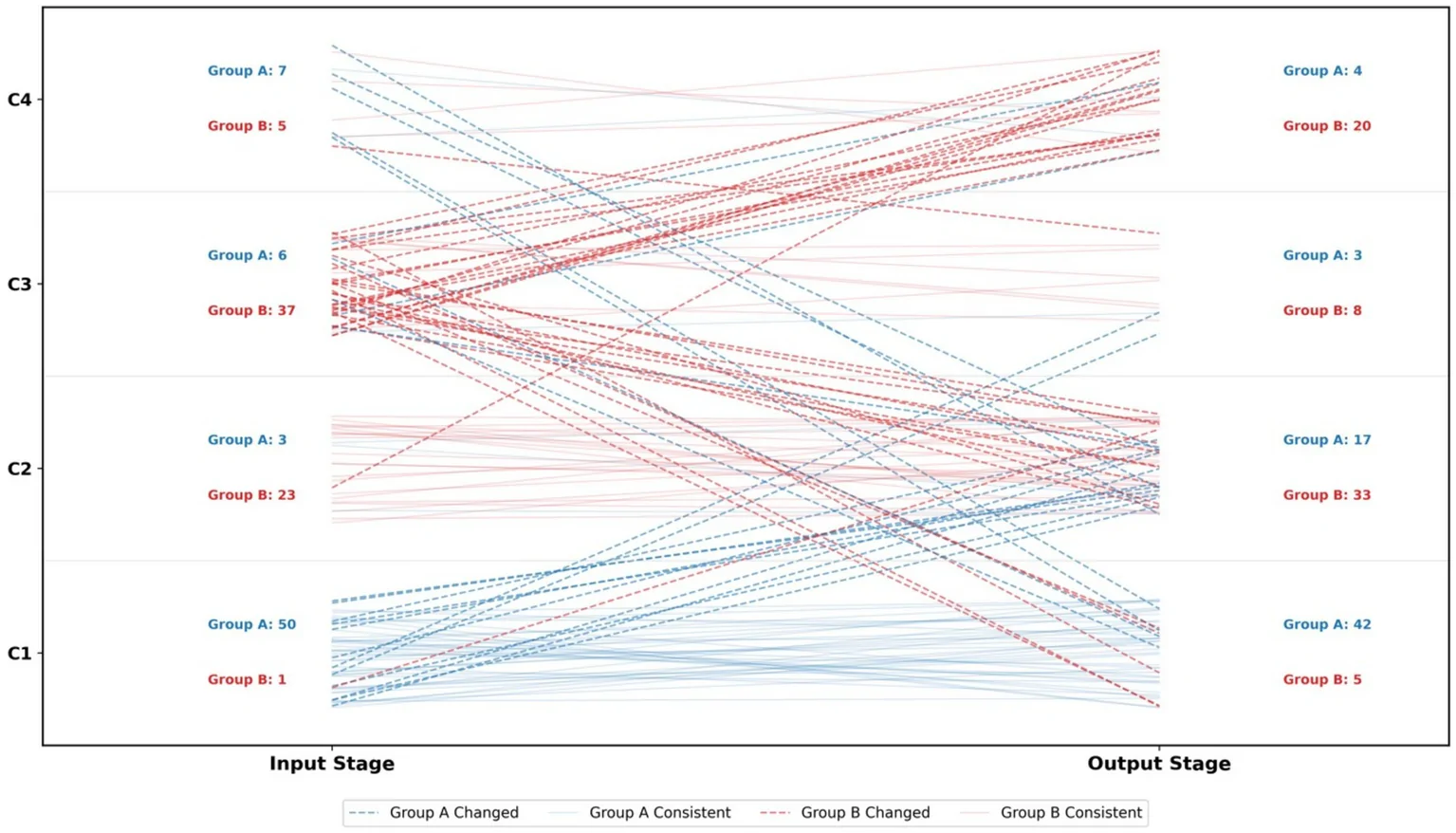
Stage transitions of interaction patterns: CACI training effect on behavioral consistency.

As shown in Figure 3, task mode and interpreting direction had little impact on the clustering results, with near-even distribution across modes and directions. By contrast, CACI training experience (the first two columns in Figure 3) shows a clear effect, with a marked imbalance between the two groups across cluster-stage combinations. The transition status by cluster and group is thus presented in Figure 4. Group A (trained students) demonstrated a higher consistency rate (66.7%) than Group B (untrained students, 50.0%). During input, Group A dominated Cluster 1 (Intensive Engagers), with 20% later transitioning to Cluster 2 (Fast Scanners) during output. Group B initially distributed across Cluster 2 (Fast Scanners) and Cluster 3 (Traditionalists), with Fast Scanners remaining stable but Traditionalists transitioning widely. Cluster 4 (Frequent Switchers) included both groups during input but turned dominated by Group B during output.

### 4.3 Associations between interpreting products and interaction patterns

The overall high transition rate (58.3%) indicates noticeable interaction reconfiguration across interpreting stages. Consequently, we examined the effects of stage-based interaction patterns on interpreting products separately (Table 6).

**Table 6:** Cluster comparisons of interpreting products.

Measures	Cluster 1	Cluster 2	Cluster 3	Cluster 4	F/H Statistics
Input-based					
Target speech duration (ms)	125,556 (48364)	119,707 (40027)	120,763 (40422)	109,171 (32177)	H = 0.94
Pause count	50.47 (26.74)	44.08 (21.12)	45.51 (19.40)	42.50 (16.98)	H = 1.60
Pause duration proportion	0.24 (0.09)	0.23 (0.10)	0.22 (0.12)	0.21 (0.06)	*F* = 0.53
Filler count	5.69 (8.53)	2.00 (1.90)	4.19 (5.93)	2.92 (1.78)	H = 1.43
Disfluencies	2.92 (3.45)	2.39 (2.21)	1.93 (1.74)	1.33 (1.30)	H = 2.91
InfoCom	6.24 (1.00)	6.48 (0.59)	6.26 (0.63)	6.71 (0.62)	H = 5.06
FluDel	5.46 (1.14)^a^	6.17 (0.92)^b^	6.08 (0.75)^b^	6.46 (0.62)^b^	H = 15.03**
TLQual	5.70 (1.04)^a^	6.35 (0.85)^b^	6.28 (0.72)^b^	6.67 (0.58)^b^	H = 18.31***
Output-based					
Target speech duration (ms)	122,227 (47638)	124,253 (43264)	135,025 (37038)	107,335 (31439)	H = 4.70
Pause count	47.26 (25.70)	48.08 (21.85)	52.55 (21.82)	41.00 (17.75)	H = 2.66
Pause duration proportion	0.23 (0.09)	0.24 (0.10)	0.29 (0.11)	0.20 (0.10)	*F* = 2.28
Filler count	5.75 (8.76)	3.12 (3.84)	2.18 (2.71)	4.46 (6.45)	H = 1.06
Disfluencies	2.93 (3.49)	2.22 (1.95)	1.36 (2.20)	1.92 (1.74)	H = 4.36
InfoCom	6.30 (1.05)	6.46 (0.62)	6.14 (0.51)	6.23 (0.63)	H = 3.99
FluDel	5.56 (1.14)	6.09 (0.89)	6.09 (1.18)	6.04 (0.67)	H = 6.84
TLQual	5.83 (0.97)	6.27 (0.86)	6.18 (1.19)	6.25 (0.73)	H = 7.32

Using interaction patterns identified at the input stage as grouping variables, only scores for fluency of delivery and target language quality showed strong pattern-related differences. *Post hoc* tests indicated that for both fluency and target language quality, significant differences emerged between Cluster 1 (Intensive Engagers) and all the other three clusters. The temporal speech measures and information completeness scores did not show significant pattern-related differences. When using output-stage behavioral patterns as grouping factors, none of the temporal speech measures or quality measures differed significantly across clusters.

## 5 Discussion

This study set out to investigate student-AI interaction patterns in CACI with eye-tracking, pen-recording, and voice-recording data collected from 22 interpreting trainees, divided into two groups based on prior CACI training experience. In summary, the analysis revealed four interpretable interaction patterns: Intensive Engagers (heavy reliance on AI), Fast Scanners (scanning-based processing), Traditionalists (very limited AI reliance), and Frequent Switchers (shifts between AI support and handwritten notes). Among these profiles, Fast Scanners (Cluster 2) demonstrated the most stable pattern across the two stages of CACI (96.2% consistency), while Traditionalists (Cluster 3) showed the greatest variability (18.6% consistency). CACI training appeared to facilitate pattern stability—trained students (Group A) demonstrated a higher consistency rate (66.7%) than untrained students (Group B, 50%). Notably, behavioral patterns at the input stage predicted interpretation quality outcomes—specifically fluency and target language quality—with Intensive Engagers (Cluster 1) showing lower fluency and quality scores than other patterns, whereas output-stage patterns showed no significant product differences. These findings highlight the heterogeneity of student-AI interaction patterns, demonstrate training effects on behavioral consistency, and reveal associations between interaction profiles and interpreting performance.

### 5.1 Heterogeneity of student-AI interaction patterns in complex bilingual tasks

The cluster analysis revealed four interpretable behavioral profiles—Intensive Engagers, Fast Scanners, Traditionalists, and Frequent Switchers—each characterized by unique configurations of interaction pathways and cognitive routes.

On the one hand, this heterogeneity of student-AI interaction patterns aligns with prior research in educational contexts such as written translation (Cai and Tian, 2026), academic writing (Nguyen et al., 2024), and programming (Güner and Er, 2025), where a spectrum of AI reliance and engagement from minimal to extensive have been documented. On the other hand, due to the inherent temporal and cross-linguistic pressure in CACI, these patterns manifest differently from those in self-paced tasks. Rather than being captured through conversational turn-taking (Chen et al., 2026) or interaction duration (Cai and Tian, 2026), CACI interaction patterns emerge through subtle differences in visual attention allocation, information retrieval strategies, and underlying processing routes.

Among the identified behavioral profiles, Intensive Engagers displayed the highest AI reading proportion and concentrated their note-taking within the AI display area, yet their processing depth remained moderate despite relatively high regression rates. Interview data revealed that this AI-centric, regressive pattern stemmed from an intensive monitoring and verification strategy, where cognitive effort primarily focused on real-time evaluation of ASR accuracy and MT quality. This pattern diverges markedly from Cai and Tian’s (2026) Reflective Deep Engagers who achieved deeper AI integration and better performance in written translation through recursive inquiry and refinement. For Intensive Engagers in interpreting, the inherent temporal demand may have precluded such deep engagement, shifting their attention toward immediate accuracy judgements. This suggests that deep, reflective AI engagement may conflict with the temporal constraints of bilingual tasks such as interpreting.

Conversely, Traditionalists maintained minimal AI engagement, relying instead heavily on short-term memory and self-generated notes, as evidenced by high deep-fixation proportions on notepads. Interview data revealed that this avoidance stemmed from profound apprehension about ineffective human-AI collaboration, echoing concerns about AI reliability (e.g., Güner and Er, 2025; Haindl and Weinberger, 2024) and technology anxiety (Zhong and Ma, 2025) documented in other educational contexts. Coupled with CACI’s severe temporal demand, which many emphasized made AI engagement too risky, such skepticism may thus reinforce their preference for established, human-centric workflows in high-stakes interpreting contexts.

Between these extremes, Fast Scanners engaged in rapid, shallow, and fragmented scanning across visual elements, while Frequent Switchers demonstrated active integration through frequent switching between AI and notepad areas. Notably, the substantial within-cluster variability in AI engagement for Fast Scanners suggests that this superficial scanning style operates independently of resource preference: they may focus on AI outputs, their own notes, or both, yet all exhibit shallow processing rather than deep engagement. By contrast, Frequent Switchers adopted a more balanced approach, using AI outputs as supplementary support for their own cognitive processing, which resembles AI-Assisted Code Refiners in Güner and Er (2025) focusing on programming tasks and Pro-active Learners in Stojanov et al. (2024) examining students’ use of ChatGPT for learning purposes. These findings together highlight that heterogeneity in student-AI interaction during interpreting is multifaceted, extending beyond AI reliance variations, and must account for distinct processing strategies and task-specific adaptations.

### 5.2 Dynamic transitions of interaction patterns and training effects

While existing studies primarily identify overall student-AI interaction patterns (Cai and Tian, 2026; López-Pernas et al., 2025; Nguyen et al., 2024), few examine pattern shifts across task stages. Those exploring temporal dynamics often focus on semester-long trajectories (e.g., Chen et al., 2026) or intervention-induced shifts (e.g., Güner and Er, 2025). The present study, however, reveals substantial within-task variability with 41.7% of participants exhibiting pattern shifts across task stages, highlighting the adaptive nature of student-AI interaction and the need for stage-based analysis in real-time task environments.

From a cluster perspective, Fast Scanners showed high stability (96.2%). This persistent, fragmented scanning likely reflects habitual processing or attention control difficulties under tight time pressure (as noted in Defrancq and Fantinuoli, 2021; Jiang, 2025; Yuan and Wang, 2023). Traditionalists, conversely, displayed the lowest stability (18.6%), often transitioning to AI-reliant or integrative patterns during output. Interestingly, the divergence between these students’ conscious intention to avoid AI—often perceiving it as disruptive or even unethical as revealed in post-task interviews—and their actual behavior shifted adaptively toward AI use during production, suggests that task demands might have overridden initial resistance.

From a training perspective, trained interpreters (Group A) exhibited higher overall pattern consistency (66.7%) than untrained students (50.0%). This enhanced stability suggests that training fosters more predictable and stable AI interaction strategies, likely due to clearer understanding of their own competence and optimal AI collaboration mode in the task (Chen et al., 2026; Güner and Er, 2025; Yang et al., 2025).

Among untrained interpreters (primarily comprised Fast Scanners and Traditionalists), some Traditionalists shifted to the most contrasting profile: Intensive Engagers. Without CACI training, these students initially deliberately avoided AI (as revealed in post-task interviews), but persistent AI content visibility during output proved difficult to resist. When facing speech production challenges, many chose to consult AI references. This delayed AI incorporation, especially under time pressure, likely induced decision-making vacillation and cognitive overload from reconciling multi-source information (i.e., self-generated notes and unfamiliar AI outputs). The resulting processing inefficiency may explain why the late-stage transitions, despite leveraging more resources, yielded no performance advantages.

Trained interpreters who were Intensive Engagers during input tended to reduce AI reliance during output. This likely reflects a strategic disengagement from external aids during task execution, a pattern also observed among AI-assisted writers (Yang et al., 2025). Alternatively, this could stem from the avoidance of interference from competing visual demands from AI outputs (ASR transcripts, MT references). As observed in sight translation, students often look away from source text during target speech production, as they found it difficult to resist linguistic interference (Chmiel and Lijewska, 2019). Similarly, trained interpreters may reduce AI consultation to minimize visual and linguistic interference, thereby preserving cognitive resources for target speech generation.

### 5.3 Performance associations with interaction patterns

Analysis of interpreting products revealed that input-stage interaction patterns have lasting consequences for output quality, whereas production-stage patterns appear less critical. This asymmetry appears different from Chen and Kruger (2024b), who reported that MT dwell time during the output phase of CACI correlated positively with interpreting quality in L2–L1 interpreting. This discrepancy may stem from workflow differences: their production relied on respeaking-based ASR output together with MT where no note-taking was involved, making MT reference a direct resource for linguistic scaffolding. In the present workflow, output depended more heavily on the mental representations and notes established during input, with AI support serving as one of several available resources. The findings therefore suggest that the influence of output-stage AI interaction is conditional on its functional role: it may be consequential when it directly supports target-language production but less so when production is constrained primarily by resources established during input.

Moreover, this finding resonates with Gile’s (2002, 2025) Effort Model, which posits that the input phase, involving intense competition among Efforts to fulfill cognitive and mechanical task demands, is crucial for determining interpreting quality. The cognitive foundation (e.g., a mental representation of the source) is also built during this stage through active listening, memory encoding, and note-taking. Conversely, the output phase is characterized by “cognitive cooperation between Efforts” (Gile, 2025, Slide 14) and mainly involves leveraging the pre-processed representations, which may exert less impact on overall interpreting quality. Empirical research on traditional consecutive interpreting provides further evidence that ear-pen span, a measure of input processing efficiency, negatively correlates with interpretation quality (Chen, 2020). Our results similarly indicate that in CACI, cognitive processing relevant to interpreting quality is predominantly determined during input, reflecting the constraining influence of input-stage processing: even optimal AI interaction strategies during output may not fully compensate for inadequate input processing.

The performance disadvantage of Intensive Engagers in interpreting fluency and target language quality is particularly noteworthy. This disadvantage may stem from the inherent challenges of multimodal processing and increased coordination demands when managing external AI-generated input during interpreting (e.g., Wang and Wang, 2019; Ünlü and Doğan, 2024; Yu and Shao, 2025), thereby undermining target speech organization and delivery of fluency and, in turn, affecting target language quality.

Moreover, this pattern could also reflect inadequate listening comprehension. While this pattern superficially resembles Reflective Deep Engagers in translation tasks who achieved superior performance (Cai and Tian, 2026), the temporal constraints of CACI might have limited participants’ ability to integrate AI outputs deeply. As indicated by their interview data and moderate regression rates, these students were constantly verifying AI-generated information. They might have anchored comprehension to AI-generated text rather than integrating it with their own cognitive processing. Therefore, while ASR and MT support allowed them to generate comparably complete content as other clusters, much of their effort appears to be consumed by content-related processing and verification, leaving fewer resources for fluent delivery and high-quality target language output. Overall, these findings align with concerns on AI over-reliance in interpreting studies about superficial comprehension and influent renderings (Cheung and Li, 2022; Defrancq and Fantinuoli, 2021).

Conversely, Frequent Switchers, who demonstrated dynamic coordination between external AI support and internal cognitive processing, achieved the best performance. Rather than treating AI output as a substitute for their own efforts, Frequent Switchers appeared to use it strategically as a complementary resource, actively cross-referencing AI suggestions against their own evolving understanding while maintaining autonomy during the interpreting process. This balanced approach leverages AI strengths while preserving human agency and critical evaluation (Gomez et al., 2025), a synergy frequently found successful in educational settings (Güner and Er, 2025; Gruenhagen et al., 2024; Jose et al., 2025; Ninghardjanti et al., 2025; Yang et al., 2025).

Interestingly, Fast Scanners and Traditionalists achieved comparable intermediate performance levels that did not differ significantly from Frequent Switchers. Fast Scanners’ rapid, shallow scanning might have allowed them to quickly extract key information from multiple sources without overinvesting in verification, while Traditionalists’ deep processing of self-generated notes serving as a highly personalized external memory (Albl-Mikasa, 2008)—though more cognitively demanding—ensured thorough comprehension. This finding suggests that multiple pathways to adequate performance exist in CACI, though the actively integrative strategy of Frequent Switchers appears most robust.

The lack of significant differences in information completeness across patterns is also noteworthy. Mean scores for all clusters exceeded 6 out of 8, indicating that all four interaction approaches enabled essential content capture. This might be due to the moderate material length, which created a ceiling effect for content retention. However, another possibility is that ASR/MT outputs, self-generated notes, and memory served as partially substitutable resources for information retention. The soft constraints hypothesis proposes that people can pursue the same task goal through different configurations of internal and external resources, selected according to their relative costs and benefits (Gray and Fu, 2004; Gray et al., 2006). This account fits the observed profiles, which differed substantially in their reliance on these resources but showed no detectable differences in information completeness, indicating that these configurations may have maintained comparable access to the source content. However, since the compensatory processes underlying this mechanism was not directly measured, this interpretation remains tentative.

While information completeness assesses whether message content is conveyed, fluency and target-language quality assess smooth delivery and grammatical, idiomatic production. The latter depend on both access to content units and a coherent, accessible representation formed during input. Extending Gile’s (2025) Human-Machine Interaction Effort proposal to CACI, intensive AI reading and verification during input may add demands to listening, memory, note-taking, and coordination. This could leave individual content units accessible while weakening their integration for later reformulation. Consistently, input-stage Intensive Engagers received significantly lower fluency and target-language quality scores, whereas output-stage patterns did not differ.

## 6 Implications and conclusions

This study examined student-AI interaction patterns in computer-assisted consecutive interpreting, a prototypical complex bilingual task with intense temporal pressure and cross-linguistic switching demands. Using triangulated eye-pen-voice data, we identified four interpretable behavioral patterns, traced their transitions across interpreting stages, examined training effects on pattern adoption, and explored connections between cluster adoption and task performance. These patterns should be interpreted as exploratory and context-bound behavioral patterns rather than stable participant types since 264 stage-level observations were nested within only 22 participants, which do not compensate for the limited participant-level sample. Despite the small sample size, this study provides key implications for AI integration in interpreter training and broader complex bilingual task instruction.

First, the heterogeneity of interaction patterns suggests that students approach AI assistance in fundamentally different ways, and pedagogical interventions may account for individual differences. Rather than prescribing a single “correct” approach, instructors could guide students to develop their own interaction strategy. Moreover, the implicitness of student-AI interaction patterns in interpreting (hidden in subtitle attention distribution and processing routes) necessitates instructors guiding students to accurately describe and actively reflect on their experience and strategy, thereby enabling more targeted instruction.

Such reflection exercises are also important since interaction patterns shift as tasks evolve. Promoting metacognitive awareness of strategy utilization enables students to critically evaluate why they adopted specific approaches at different task stages and whether alternatives are more effective. It also helps students develop adaptive expertise necessary for effective AI-assisted interpreting across diverse contexts.

Furthermore, the negative performance associations shown by the Intensive Engagers highlight the importance of training students to maintain critical in AI engagement. Instruction should emphasize that AI-generated text is a resource to be evaluated and integrated, not a script to be followed. Encouraging students to identify AI errors, compare AI suggestions with their own rendering, and make explicit decisions about when to leverage AI support can cultivate evaluative skills necessary for human-AI collaboration.

Meanwhile, the performance advantages associated with the Frequent Switchers suggest that instead of considering AI support and traditional note-taking as mutually exclusive, students should develop synergistic workflows. This could involve explicit instruction on using AI transcripts for specific purposes (e.g., capturing proper nouns or numerical data) while maintaining conventional note-taking for comprehension facilitation and speech organization.

Finally, the stage-specific nature of interaction patterns has implications for interface design. CACI platforms should offer tailored display configurations or interaction affordances for input versus output stages. For example, during input, interfaces can enhance ASR transcript clarity through strategic formatting (e.g., spacing within meaning chunks, highlighting low-confidence segments) for rapid information filtering. During output, interfaces could simply facilitate flexible access to multiple resources while minimizing visual interference.

This study, while offering valuable insights, is subject to several limitations. The relatively small sample and its restriction to top-tier interpreting programs in China may limit the generalizability of the findings. Moreover, recruiting CACI-trained participants from one institution and untrained participants from three others makes it difficult to separate training effects from institutional influences, including differences in participants’ academic and socio-cultural contexts. Future research should employ larger, more diverse samples and carefully designed multi-institutional comparisons. Additionally, while our study examines training-related differences cross-sectionally, longitudinal studies are needed to trace how student-AI interaction patterns evolve over time.

## Data Availability

The original contributions presented in the study are included in the article/Supplementary material, further inquiries can be directed to the corresponding author.
