# Supplementary material for: Student-AI interaction in computer-assisted consecutive interpreting: patterns and performance
Source: Front Psychol. 2026 Sep 11;17:1890808. doi: 10.3389/fpsyg.2026.1890808 (PMC13612447; doi:10.3389/fpsyg.2026.1890808)
Supplement: Supplementary file 1 [file Supplementary_file_1.docx]

**Appendices**

**Appendix A: Calculation of Material-Comparability Measures**

The six experimental speeches were examined in terms of presentation duration, information density, sentence length, lexical frequency, and dependency-based syntactic complexity. Table A1 summarizes the operational definitions and language-level descriptive results. The item-level values are reported in Table 2 in the manuscript.

**Table A1** Summary of Material-Comparability Measures for the English and Chinese Speech Sets

| **Measure** | **Operational definition** | **English, M (range)** | **Chinese, M (range)** |
| --- | --- | --- | --- |
| Duration (s) | Recorded presentation time | 81.7 (76–88) | 82.3 (78–85) |
| Idea density | Propositions divided by word count | .504 (.500–.508) | .507 (.504–.511) |
| Sentence length | Word count divided by sentence count | 12.94 (11.56–13.89) | 12.37 (11.33–13.50) |
| Zipf frequency | Mean SUBTLEX frequency of content-word forms | 3.97 (3.82–4.09) | 4.51 (4.37–4.62) |
| Dependency distance | Mean dependent–head positional distance | 2.33 (2.27–2.40) | 2.51 (2.32–2.66) |
| SUBTLEX coverage (%) | Content-word tokens represented in the frequency database | 96.89 (93.33–98.78) | 97.54 (94.34–100.00) |

*Note*. Values are means and ranges across the three speeches in each language. Lower Zipf values indicate less frequent word forms. SUBTLEX coverage is reported as a methodological check rather than as a measure of lexical difficulty.

English idea-density values were calculated using the Computerized Propositional Idea Density Rater 5.0 (CPIDR 5.0). For Chinese, HanLP-assisted semantic-role analysis was first used to identify potential predicate–argument structures. These candidates were then manually coded using rules informed by Chinese Proposition Bank 3.0. The coding considered predicates and secondary predicates, as well as relations expressing location, time, manner, adverbial modification, discourse connection, direction, topic, scope, purpose or cause, coordination, beneficiary, and condition. The final proposition counts were manually reviewed before being divided by the segmented-word counts. In both languages, idea density was calculated by dividing proposition count by word count.

Lexical difficulty was operationalized as the mean Zipf frequency of content-word forms. Universal POS tags were used to identify nouns, verbs, adjectives, and adverbs, while proper nouns, numerals, auxiliaries, function words, and punctuation were excluded. English word-form frequencies were obtained from SUBTLEX-US, and Chinese word-form frequencies from SUBTLEX-CH. Frequency per million was converted to the Zipf scale, and the mean was calculated across nouns, verbs, adjectives, and adverbs represented in the corresponding database. Proper nouns, numerals, auxiliaries, function words, punctuation, and word forms not represented in the database were excluded. Database coverage ranged from 93.33% to 100.00% across the six speeches.

The English and Chinese speeches were tokenized, POS-tagged, and dependency-parsed locally using UDPipe 1.4.0.1 under the Universal Dependencies framework. The English-EWT and Chinese-GSDSimp UD 2.5 models (release 191206) were used for the respective languages. Dependency distance was defined as the absolute linear distance between each dependent and its syntactic head. Punctuation dependencies and root relations were excluded, and mean dependency distance was calculated across all remaining dependency relations within sentence boundaries.

**Appendix B	: Details of Cluster Analysis**

This appendix provides supplementary materials for the cluster analysis reported in the Results section. Table B1 reports internal validity metrics used to determine the optimal number of clusters from *k* = 2 to *k* = 8. Table B2 presents cluster centroids based on combined stage data. Figure B1 displays the principal component analysis (PCA) projection of the four cluster-solution, both combined and separated by stage. Table B3 summarizes the operational basis of the profile labels and the neutral descriptors considered during revision. Table B4 and Figure B2 shows how the four reported patterns were represented in the three-cluster and five-cluster solution. Table B5 reports the robustness analyses.

**Table B1** Internal Validity Metrics for Cluster Solutions

| k | **Silhouette Coefficient** | **Calinski-Harabasz Index** | **Gap Statistic** | **Combined Score** |
| --- | --- | --- | --- | --- |
| 2 | 0.240 | 100.76 | 0.679 | 0.333 |
| 3 | 0.248 | 92.13 | 0.730 | 0.381 |
| **4** | **0.268** | **88.99** | **0.790** | **0.664** |
| 5 | 0.257 | 86.85 | 0.856 | 0.587 |
| 6 | 0.255 | 81.62 | 0.881 | 0.534 |
| 7 | 0.248 | 77.36 | 0.895 | 0.416 |
| 8 | 0.244 | 74.99 | 0.926 | 0.379 |
| *Note*. Each metric was min-max normalized across k values. The three normalized metrics were then averaged to produce the combined score. The selected solution (k = 4) is shown in bold. | | | | |

**Table B2** Cluster Centroids for the Four-Cluster Solution

| **Cluster** | **AI Reading Proportion** | **Regression Rate** | **Saccade Amplitude** | **Fixation Frequency (per min)** | **Deep Fixation Proportion** | **Switch Count** |
| --- | --- | --- | --- | --- | --- | --- |
| C1 | 0.83 | 0.33 | 3.24 | 165.43 | 0.39 | 3.30 |
| C2 | 0.27 | 0.27 | 2.20 | 218.61 | 0.17 | 7.00 |
| C3 | 0.12 | 0.32 | 3.87 | 103.36 | 0.56 | 7.65 |
| C4 | 0.35 | 0.33 | 4.14 | 163.40 | 0.39 | 36.03 |
| *Note.* Values represent unstandardized cluster centroids. AI reading proportion and deep fixation proportion (>250 ms) are bounded [0, 1]. Fixation frequency is expressed as fixations per minute. Switch count represents total transitions between the AI interface and notepad. Raw numerical cluster labels were reordered solely for consistent presentation. This relabelling did not alter cluster membership. | | | | | | |

**Figure B1** Principal Component Analysis (PCA) Projection of the Four Clusters: Combined Data (left) and Separated by Stage (right)

*Note.* Stars (★) indicate cluster centroids. PC1 and PC2 collectively explain 62.2% of total variance.

**Table B3** Operational Basis of the Pattern Labels

| **Pattern Labels** | **Defining Centroid Configuration** | **Supplementary Descriptive Evidence** | **Neutral Descriptor Considered** |
| --- | --- | --- | --- |
| Intensive Engagers | Highest AI-reading proportion and low switching | Greatest AI-area note-taking and limited notepad use | AI-focused low-switch readers |
| Fast Scanners | Highest fixation frequency; lowest regression rate, saccade amplitude, and deep-fixation proportion | Note-taking measures were used descriptively but did not uniquely define this profile | Rapid shallow scanners |
| Traditionalists | Lowest AI-reading proportion and fixation frequency; highest deep-fixation proportion | Greatest descriptive notepad-based note-taking and minimal AI-area note-taking | Note-focused low-AI processors |
| Frequent Switchers | Exceptionally high switch count, with moderate AI reading | The label was based primarily on frequent transitions between the AI display and notepad | High-switch integrators |

*Note.* Pattern labels were assigned after clustering and did not influence the selection of *k* or cluster membership. Note-taking measures and cued-retrospection interviews were used for descriptive and explanatory triangulation rather than as clustering variables or independent validation criteria.

Although the three-cluster solution remained interpretable, it did not adequately separate two substantively different interaction configurations. Moving from *k* = 3 to *k* = 4 reduced the standardized within-cluster sum of squares from 928.50 to 781.52, a reduction of 15.8%. As shown in Table B4, the three-cluster solution largely preserved the Intensive Engager and Fast Scanner patterns but combined most Traditionalist and Frequent Switcher observations into the same cluster.

**Table B4** Sensitivity Analysis of the Retained Cluster Solution

| **Pattern in the retained solution** | **n** | **Distribution in k=3** | **Distribution in k=5** |
| --- | --- | --- | --- |
| Intensive Engagers | 98 | AI-focused: 97 (99.0%); merged low-AI/high-switch: 1 (1.0%) | Intensive Engager-like: 75 (76.5%); AI-focused fast-scanning hybrid: 23 (23.5%) |
| Fast Scanners | 76 | Rapid scanning: 75 (98.7%); merged low-AI/high-switch: 1 (1.3%) | Fast Scanner-like: 47 (61.8%); AI-focused fast-scanning hybrid: 29 (38.2%) |
| Traditionalists | 54 | Merged low-AI/high-switch: 49 (90.7%); AI-focused: 3 (5.6%); rapid scanning: 2 (3.7%) | Traditionalist-like: 48 (88.9%); Intensive Engager-like: 4 (7.4%); Fast Scanner-like: 1 (1.9%); Frequent Switcher-like: 1 (1.9%) |
| Frequent Switchers | 36 | Merged low-AI/high-switch: 31 (86.1%); rapid scanning: 3 (8.3%); AI-focused: 2 (5.6%) | Frequent Switcher-like: 36 (100.0%) |

*Note.* Entries show the number and within-row percentage of observations assigned to each cluster under the alternative solutions. Descriptive cluster names for the three- and five-cluster solutions are used only to facilitate comparison and do not represent additional formally interpreted patterns.

The cross-solution mapping reveals different limitations of the adjacent solutions. The three-cluster solution largely preserved the Intensive Engager and Fast Scanner profiles but combined most Traditionalist and Frequent Switcher observations, thereby obscuring the distinction between low-AI, note-centred processing and high-switch resource integration. In contrast, the five-cluster solution largely preserved the Traditionalist and Frequent Switcher profiles but subdivided the Intensive Engager and Fast Scanner profiles. Its additional cluster combined 23 Intensive Engager observations and 29 Fast Scanner observations and was characterized by relatively high AI reading together with rapid, shallow scanning. Thus, the fifth cluster represented a finer subdivision along the boundary between two existing profiles rather than a profile required to distinguish the major behavioural configurations.

Although the five-cluster solution reduced within-cluster sum of squares by 13.4% relative to the four-cluster solution, such a reduction is expected when an additional cluster is introduced. The five-cluster solution showed a lower silhouette coefficient (0.257 vs. 0.268), Calinski–Harabasz index (86.85 vs. 88.99), and combined score (0.587 vs. 0.664). The four-cluster solution therefore provided a more parsimonious balance between statistical separation and substantive interpretability.

**Figure B2** Cluster-Membership Correspondence under Competing Solutions

*Note.* Band widths represent observation counts. Colours correspond to the four profiles in the retained four-cluster solution. Pattern-like descriptions for the three- and five-cluster solutions are used only to facilitate cross-solution interpretation.

**Table B5** Robustness of the Four-Cluster Solution

| **Robustness analysis** | **Replications** | **Median ARI [2.5th, 97.5th percentile]** | **All nine defining characteristics reproduced** |
| --- | --- | --- | --- |
| Different random seeds | 100 | 0.854 [0.809, 0.976] | 100% |
| Leave-one-participant-out refitting | 22 | 0.927 [0.729, .0985] | 100% |
| Subsampling 18 of 22 participants | 300 | 0.768 [0.481, 0.935] | 80.0% |
| Participant-level bootstrap | 300 | 0.681 [0.390, 0.880] | 62.3% |

*Note.* ARI = adjusted Rand index; an ARI of 1 indicates identical membership. All refits used 10 initializations. For participant-level resampling, participants rather than individual observations were resampled, and the six variables were re-standardized within each resampled dataset. Cluster labels were aligned with the reported solution before ARI was calculated. The nine defining characteristics refer to the centroid-ordering features used to operationalize the four profiles.

Collectively, these analyses indicate that the defining centroid configurations were more reproducible than the exact assignment of every task observation. The four patterns are therefore interpreted as an exploratory behavioural typology rather than as a fixed participant taxonomy.
